# Superhydrophilic Coating with Antibacterial and Oil-Repellent Properties via NaIO_4_-Triggered Polydopamine/Sulfobetaine Methacrylate Polymerization

**DOI:** 10.3390/polym12092008

**Published:** 2020-09-03

**Authors:** Hsiu-Wen Chien, Hong-Yu Lin, Chau-Yi Tsai, Tai-Yu Chen, Wei-Nian Chen

**Affiliations:** 1 Department of Chemical and Materials Engineering, National Kaohsiung University of Science and Technology, Kaohsiung 000807, Taiwan; 1105101163@gm.kuas.edu.tw (H.-Y.L.); 1105311142@nkust.edu.tw (T.-Y.C.); 2 Photo-Sensitive Material Advanced Research and Technology Center (Photo-SMART Center), National Kaohsiung University of Science and Technology, Kaohsiung 000807, Taiwan; 3 Department of Materials Engineering and Science, National Formosa University, Yunlin County 000640, Taiwan; cytsai503@nfu.edu.tw (C.-Y.T.); 10772109@gm.nfu.edu.tw (W.-N.C.)

**Keywords:** polydopamine, sulfobetaine methacrylate, superhydrophilic, antifouling

## Abstract

Superhydrophilic coatings have been widely used for the surface modification of membranes or biomedical devices owing to their excellent antifouling properties. However, simplifying the modification processes of such materials remains challenging. In this study, we developed a simple and rapid one-step co-deposition process using an oxidant trigger to fabricate superhydrophilic surfaces based on dopamine chemistry with sulfobetaine methacrylate (SBMA). We studied the effect of different oxidants and SBMA concentrations on surface modification in detail using UV–VIS spectrophotometry, dynamic light scattering, atomic force microscopy, X-ray photoelectron spectroscopy, and surface plasmon resonance. We found that NaIO_4_ could trigger the rate of polymerization and the optimum ratio of dopamine to SBMA is 1:25 by weight. This makes the surface superhydrophilic (water contact angle < 10°) and antifouling. The superhydrophilic coating, when introduced to polyester membranes, showed great potential for oil/water separation. Our study provides a complete description of the simple and fast preparation of superhydrophilic coatings for surface modification based on mussel-inspired chemistry.

## 1. Introduction

The surface modification of polymeric materials is the primary step for improving their surface properties to extend their utility [1,2,3,4]. Although plastic substrates offer many advantages, such as light weight, flexibility, transparency, excellent mechanical properties, and electrical and thermal insulation, the water contact angle of most plastic materials lies between hydrophobicity and hydrophilicity (10° < *θ_w_* < 90°), which can be inadequate for several applications. Thus, the modification of the surface property of polymeric materials into superhydrophobic or superhydrophilic and maintaining their bulk properties is an important field of investigation. A superhydrophilic surface generally exhibits a water contact angle lower than 10° [5,6,7]. On such a surface, water droplets spread rapidly and flow down the surface at a considerable velocity [5]. The resulting thin water film can prevent staining by oil-based pollutants and provide anti-fouling properties [8,9,10]. Thus, superhydrophilic surfaces have significant potential for practical applications in wastewater treatment, separation of oily water, optical devices, and biomedical devices. Various synthetic and natural polymers have been used in the past to produce hydrophilic surfaces.

Among them, zwitterionic polymers are attractive materials for superhydrophilic coatings [11,12]. Polysulfobetaine is a type of zwitterionic polymer. Its pendant groups, which have a quaternary ammonium cation and sulfonate anion, can retain a net neutral charge and form a strong hydration via ionic solvation [13,14]. Despite many efforts for preparing superhydrophilic surfaces using different strategies, including surface coating, surface grafting, and layer-by-layer assembly [15,16,17,18], an effective method for producing superhydrophilic surfaces is yet to be discovered, owing to the absence of functional groups in the plastic molecular skeleton.

Dopamine is an organic compound containing a catechol group and side-chain amine. It readily forms insoluble polydopamine (PDA) in alkaline solutions (tris-base, pH 8.5) via oxidative polymerization and non-covalent aggregation and then deposits on the surface targeting widespread applications, such as oil separation [19], drug release [20], sensing [21], shielding [22], membranes [23]. However, the synthesis of PDA coatings is usually time-consuming. Substrates are usually immersed in an alkaline dopamine solution for up to 12–18 h to deposit an adherent layer of PDA on the surface [24,25]. In the quest for a simple procedure, Kim et al. found that the alkaline dopamine oxidation reaction rate could be significantly increased under a pure O_2_ environment compared to the analogous reaction under ambient air [26]. Pozio et al. found that the oxidant employed, sodium periodate (NaIO_4_), could apparently complete the conversion of dopamine to o-quinone within a few minutes and accelerate the formation of thick PDA films [27]. Tahroudi et al. reported that the oxidative action of NaIO_4_ enhanced the coating speed by over 200 times [22]. Similarly, CuSO_4_/H_2_O_2_ can also enormously enhance the deposition rate of PDA coatings on substrates within 10 min compared to conventional processes [28].

Recently, dopamine was found to generate radicals in an oxidative state, which can act as initiators for the free radical polymerization of (methyl) acrylic monomers [29,30]. Sheng et al. indicated that the PDA layer on a surface can act as a photo-initiating layer to initiate the radical polymerization of a variety of (methyl) acrylic/styrene monomers under exposure to sunlight. Sunlight brings PDA to the photo-excited state to generate reactive radicals, which initiate the polymerization of monomers through a radical chain propagation mechanism [29]. Similarly, Zhang et al. proposed a reasonable mechanism for the dopamine-triggered polymerization of acrylate monomers. Under alkaline conditions, dopamine is first oxidized into dopaminoquinone and then transferred into semi-quinone radical species via a single-electron exchange reaction. These radicals act as reactive species initiating the free-radical polymerization of acrylate monomers to form polymers [30]. Zhang et al. prepared an anti-fouling surface via co-deposition of dopamine and sulfobetaine methacrylate (SBMA). However, the formation of PDA/poly (SBMA) requires 8 h, which is not effective in industrial practice.

To overcome this limitation, we used UV light and an oxidant trigger to accelerate co-deposition. In our study, the operating temperature, dopamine concentration, and deposition time were fixed at 25 °C, 2 mg/mL, and 2 h, respectively. We investigated the influence of using UV light and NaIO_4_ as a trigger and the effects of the amounts of SBMA on the surface modification in detail via UV–VIS spectrophotometry, dynamic light scattering (DLS), atomic force microscopy (AFM), X-Ray photoelectron spectroscopy (XPS), and surface plasmon resonance (SPR). Moreover, the surface wettability and antifouling performance of the modified co-depositions were also investigated. Finally, co-deposition was introduced to polyester membranes for oil/water separation applications.

## 2. Materials and Methods

### 2.1. Materials

Polyethylene terephthalate (PET) plates (Far Eastern New Century Corporation, Taipei, Taiwan) and polyester filter papers (Steritech CAT No. 1300014, Kent, WA, USA), with a diameter of 4.7 cm and pore size of 0.2 μm, were employed. Dopamine (99%) and tris(hydroxymethyl)aminomethane (Tris, 99.8%) was purchased from ACROS Organics™. SBMA was purchased from Hopax Fine Chemicals (min. 98%). All other chemicals were purchased from Sigma-Aldrich unless mentioned otherwise.

Phosphate-buffered saline (PBS) contained 137 mM NaCl (Shimakyu’s Pure Chemicals, 99.8%), 2.7 mM KCl (SHOWA, min. 99.5%), 10 mM Na_2_HPO_4_ (SHOWA, 99%), and 1.8 mM KH_2_PO_4_ (SHOWA, min. 99.5%) at pH 7.4. Trypticase soy agar contained 15 g/L agar (BD, Franklin Lakes, NJ, USA), with 15 g/L tryptone (Cyrusbioscience, Taipei, Taiwan), 5 g/L soy peptone (Cyrusbioscience, Taipei, Taiwan), and 5 g/L NaCl. The bacterial suspension buffer was prepared using 0.85% NaCl at pH 7.

### 2.2. Surface Coating of PDA/SBMA

In Table 1, we provide a detailed description of the deposition of PDA/SBMA on the substrates. The 1 × 1 cm^2^ PET plates were first cleaned with deionized (DI) water and then soaked in 1 N NaOH for 60 min. The substrates were put on the 12 well plate and then immersed in a 2 mL of reaction solution containing dopamine (2 mg/mL) and SBMA in a series of mass ratios of dopamine to SBMA from 1:0 to 1:10, 1:15, 1:25, 1:40, and 1:60 in tris buffer solution (pH = 8.5, 10 mM) for 2 h. Exposure to UV light and 5 mM NaIO_4_ also triggered the reaction. After the reaction, the substrates were removed, rinsed with deionized water (DI) water, dried, and stored at room temperature.

### 2.3. Characterization

The reaction solution at 2 h was studied using a UV-Vis spectrophotometer (ChromTech CT-2800, Taipei, TaiWan) in the wavelength range of 200–700 nm. The polymer particle size after 2 h of oxidation was characterized using DLS (90Plus Particle Size Analyzer, Brookhaven Instruments, Holtsville, NY, USA).

An SPR-T200 (Sensby Biotech Co., Ltd., Wenzhou, China) instrument was used to study the coverage of PDA/SBMA on the gold sensor slides. It was equipped with a 632.8 nm light source and an optical range of 20–80° internal reflection. All measurements were acquired on the same spot, enabling the determination of the angular variation during the measurement. Surface roughness was analyzed using AFM in tapping mode (NaioAFM, Nanosurf AG, Liestal, Switzerland).

The surface composition was measured using XPS (PHI VersaProbe, Chanhassen, MN, USA). The X-ray source was monochromatic Al-Kα (hν = 1486.6 eV) with a take-off angle of 45°. The high-resolution spectra were deconvoluted by mixing Gaussian–Lorentzian functions using the free software program, XPSPEAK. Elemental quantification was performed on the peak areas of the C1s, O1s, N1s, and S2p multiplex.

The wettability of the coating surfaces was evaluated by static water contact angle (WCA) measurements (OSA60SS, CHAO-DEES SCIENCE CO., LTD., Kaohsiung, Taiwan) with deionized water. At least six droplets (20 μL) were measured for each sample. The coatings on the surface were immersed in 0.1 M NaCl for 24 h and 7 d to test their stability. After rinsing with water and drying, the change in coating was monitored by WCA measurements.

The morphology of the membranes was examined by field emission scanning electron microscopy (FESEM, JEOL JSM-6701F, Peabody, MA, USA). Based on the SEM images (10,000×), the surface porosity of the membranes was determined using ImageJ software.

### 2.4. Non-Fouling Properties

The non-fouling properties of the surface coatings were evaluated by bacterial attachment. The test was challenged with *Escherichia coli*. The bacteria were suspended in sterilized saline at pH 7 to a final concentration of approximately 10^6^ CFU/mL. Subsequently, 1 mL of the bacterial suspension was added to the samples and incubated at 37 °C for 1 h. The samples were then rinsed with sterilized saline. The surviving bacteria on the surfaces were stained with SYTO 9 (S34854, Invitrogen, Shanghai, China) to determine bacterial attachment. After washing, live (green) bacteria on the surfaces were observed using a fluorescence microscope (Zeiss, Oberkochen, Germany). The photographs were further analyzed using Image J to quantify fluorescence intensity and evaluate bacterial attachment.

### 2.5. Water Flux

The water fluxes were calculated by measuring the time required for the flow of a constant volume of feed solutions. To describe briefly, a piece of wetted filter paper (4.7 cm in diameter) by DI water was placed in a Büchner funnel on a suction filtration setup. The height of the feed solution was fixed at 5 cm and kept flowing for 30 min to ensure the setup was in a steady state. Then, the time required was recorded by collecting 50 mL of permeate. Water flux was calculated according to the Equation:

J = Q/AT

where J is the permeation flux (L/m^2^ h), Q is the permeation volume of the testing solution (L), A is the effective area of the tested membrane (m^2^), and T is based on the time (h) of permeate water collected within a given volume.

### 2.6. Oil/Water Separation Test

The n-hexane-in-water emulsion was prepared by dissolving 0.5 g of sodium dodecyl sulfate (SDS) in 990 mL of water. Subsequently, n-hexane was added to the above mixture at a volume ratio of 1:99 for overnight stirring. The oil/water separation experiments were performed using a suction filtration setup. The membranes were pre-compacted for 5–10 min with DI water until their permeation flux stabilized. Next, the emulsion was poured onto the membrane and permeated through it. The separation results were recorded using a digital camera and optical microscopy (Zeiss, Oberkochen, Germany). The droplet sizes in the collected filtrate were measured using DLS.

## 3. Results and Discussion

### 3.1. Effect of Oxidant Triggers

**Figure 1:**
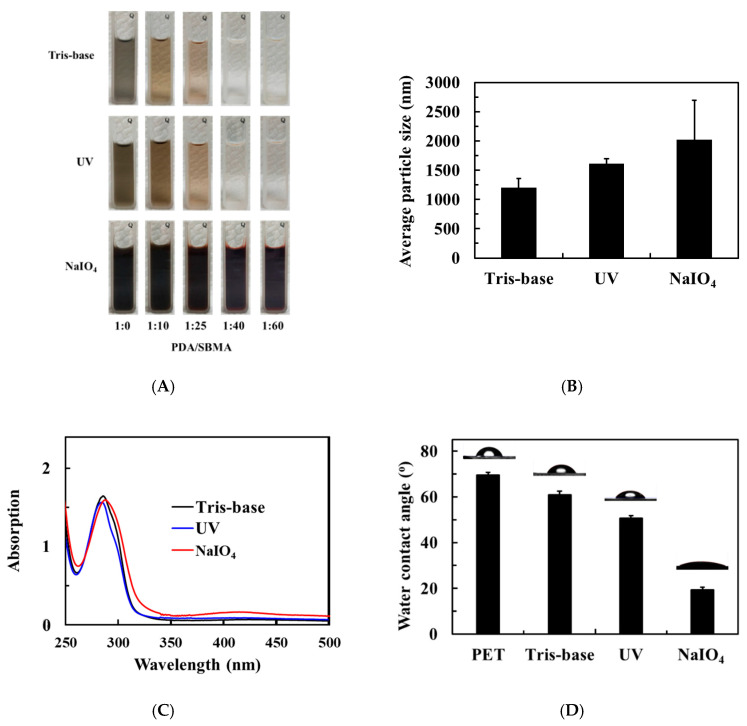
(**A**) Color changes of the various PDA/SBMA solutions in tris-base solution (pH = 8.5) with UV light and NaIO_4_ trigger. (**B**) Particle size and (**C**) UV-VIS spectra of PDA (2 mg/mL) solution at 2 h with UV light and NaIO_4_ trigger. (**D**) Water contact angles (WCAs) of the PDA coating on a polyethylene terephthalate (PET) substrate with UV light and NaIO_4_ trigger.

The color of the solution containing PDA in tris-base with UV and NaIO_4_ triggers is shown in Figure 1A. When dopamine was dissolved in tris-base, the solution was transparent at first and then gradually turned brown after 2 h of reaction. The brown color of the PDA, which results from its melanin-like structure, indicates successful dopamine self-polymerization [31]. At the same reaction time, the color of the PDA solution with an additional UV trigger becomes darker compared to the PDA solution without a UV trigger. This result implies that UV irradiation can trigger the polymerization of PDA. A previous study reported that UV irradiation can generate reactive oxygen species, such as singlet superoxide radicals (O_2_−) and hydroxyl radicals (OH), which act as oxidants required to enhance the rate of dopamine polymerization [32]. This explains the darker color of the PDA solution under UV irradiation.

The NaIO_4_-triggered PDA solution turned dark gray immediately and maintained the color until 2 h. According to the DLS results shown in Figure 1B, the particle sizes of PDA in the tris-base solutions without trigger, with UV trigger, and with NaIO_4_ trigger were 1206, 1613, and 2021 nm, respectively. In addition, spectrophotometry revealed the formation of o-quinone at around 305 nm under all reaction conditions at 2 h. However, only the NaIO_4_-triggered PDA solution exhibited an obvious band at 410 nm, indicating the formation of dopaminochrome (Figure 1C) [33]. This means that NaIO_4_-triggered solution undergoes the fastest oxidation polymerization of dopamine, followed by the UV-triggered solution, whereas that without a trigger is the slowest.

Generally, dopamine in an alkaline solution, polymerized for several hours, deposits a PDA layer in the presence of substrates, which is extremely long for practical applications. The above tests show that NaIO_4_ could trigger the polymerization of dopamine. We tried to understand the effect of UV and NaIO_4_ on the 2 h deposition of PDA. We evaluated the surface wettability of the PDA coating using WCA and the results are shown in Figure 1D. For nascent PET, the WCA was almost 69.5°. In only the tris-base solution, the WCA of the PDA-modified surfaces declined to 61.0°, which was attributed to the intrinsic hydrophilicity of PDA. Under additional UV exposure, the WCA of the PDA-modified surfaces further declined to 50.5°, indicating that UV can trigger fast oxidation polymerization and deposition. It is worth noting that NaIO_4_ can trigger rapid aggregation of polymeric products and deposition. The WCA of the PDA-modified surfaces with NaIO_4_ trigger is 19.5°. The observed value of WCA is lower than that of a previous study in which a 16 h coating on a poly(l-lactide-co-caprolactone) film changed the WCA from 74.4° to 40.3° [24].

### 3.2. Effect of the Dopamine-to-SBMA Ratio

After understanding that the additional NaIO_4_ can promote PDA polymerization and deposition, we further investigated the effect of the dopamine/SBMA ratio on the color of the solution (Figure 1A). With the dopamine solution with SBMA in the tris-base for 2 h of polymerization, we observed that the color of the solution color gradually faded as the concentration of SBMA increased. When the mass ratio of dopamine to SBMA was 1:50, the solution was almost transparent, implying that excess SBMA probably inhibits the aggregation of the polymerized products [30]. Similarly, the color of the solution gradually fades with the increase in SBMA concentration under UV-triggered conditions. Overall, the color of the solution exposed to UV was darker than that without the UV trigger. In contrast, the color of the solution for all mass ratios of dopamine to SBMA immediately turns dark gray when triggered by NaIO_4_ trigger. Since we cannot discern the depth of color with the naked eye, DLS was applied to characterize the particle sizes of the polymeric products. The results show a gradual decrease in particle size with an increase in SBMA concentration (Figure S1). The results confirmed that excess SBMA inhibits the aggregation of the polymerized products, while NaIO_4_ can also promote dopamine and SBMA polymerization.

**Figure 2:**
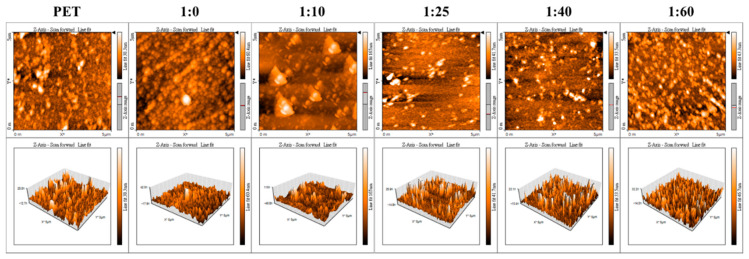
Three-dimensional (3D) atomic force microscopy (AFM) image of the various PDA/SBMA coatings on the PET substrate.

**Figure 3:**
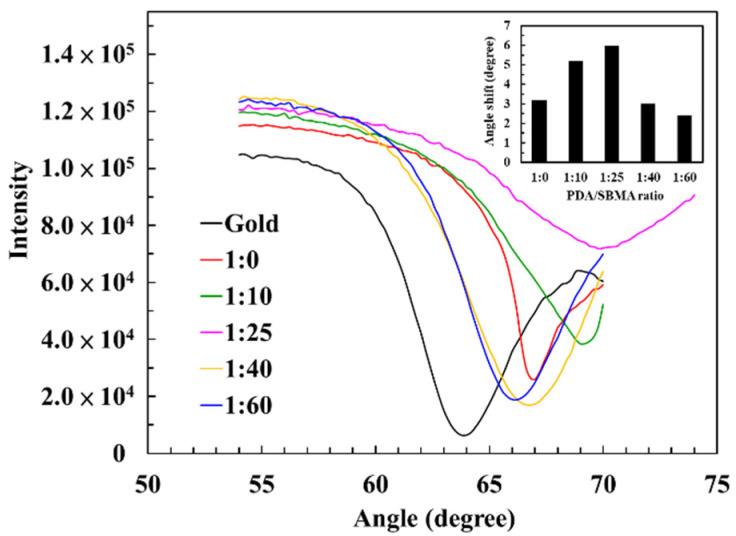
Surface plasmon resonance (SPR) curve of the various PDA/SBMA coatings. The inset shows the angular shift for the various PDA/SBMA coatings.

We further investigated the deposition of PDA/SBMA via a NaIO_4_ trigger. The surface topographies of the PDA/SBMA coatings were analyzed using AFM (Figure 2). The original PET is smooth and its root mean square (Rs) value is 3.95 nm. After coating with PDA/SBMA, we observe the deposition of polymer particles on the surfaces, which increase the Rs value. The Rs values of 1:0, 1:10, 1:25, 1:40, and 1:60 were 8.22, 6.14, 4.32, 3.47, and 5.43 nm, respectively. The increased Rs value means the surfaces became much rougher, which confirms that the PDA/SBMA was successfully bound to the surface. In order to evaluate the amount of the various PDA/SBMA coatings deposited on the gold surfaces, the full angular scans of the reflectivity spectra were acquired using SPR in PBS solution. In Figure 3, we show the SPR curves before and after the exposure of the Au slides to PDA/SBMA. We observe changes in the angles of the SPR curves, as well as the contrast of reflected intensities, for different PDA/SBMA. The angular shifts first increase and then decrease as the concentration of SBMA increases. The general trend shows that the SPR angle increases with increasing thickness of the thin film [34,35]. The largest angular shift is 1:25, which implies that the ratio has a higher packing density on the gold surface. The smallest angular shift is 1:60, which implies a thin coating.

**Figure 4:**
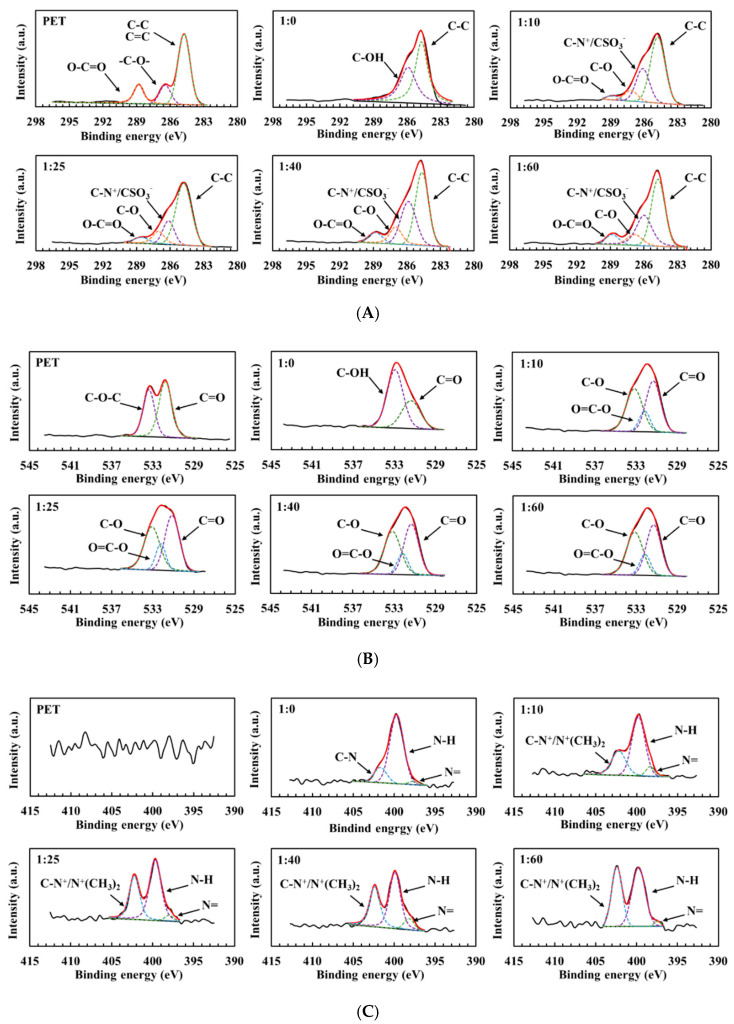

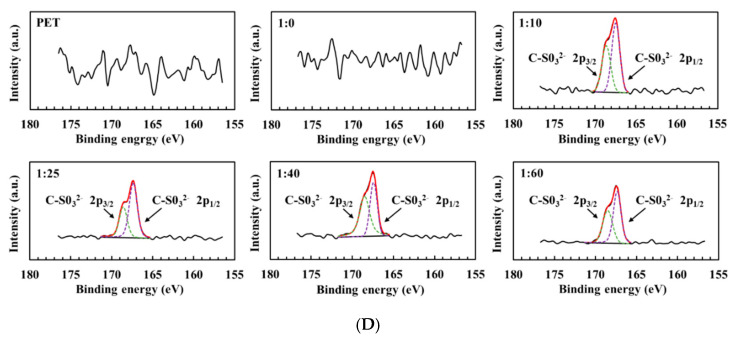
High-resolution spectra of C1s (**A**), O1s (**B**), N1s (**C**), and S2p (**D**) of the PDA/SBMA coatings.

XPS analysis was used to study the composition of the NaIO_4_ triggered PDA/SBMA coatings. The elemental compositions of carbon (C), oxygen (O), nitrogen (N), and sulfur (S) were surveyed by scanning the bonding energy from 0 to 1200 eV. First, C1s and O1s peaks were detected in the XPS spectrum of the original PET substrates (Figure 4A,B). After coating with PDA (1:0), the N1s signals appeared and the atomic percentage of nitrogen increased from 0% to 7.2%, which is attributed to the elemental nitrogen in the PDA layer (Figure 4C). SBMA contains sulfur, whereas naked PET and PDA do not; therefore, the S2p signal at 168 eV appears in the XPS spectra of the 1:10, 1:25, 1:40, and 1:60 coatings (Figure 4D). The results confirm the presence of sulfobetaine moieties on the surface. The atomic percentages of sulfur in the 1:0, 1:10, 1:25, 1:40, and 1:60 coatings were 0%, 0.9%, 1.8%, 1.6%, and 1.6%, respectively (Table 1); this indicates that, when the mass ratio of dopamine to SBMA is 1:25, a larger amount of SBMA is present.

**Figure 5:**
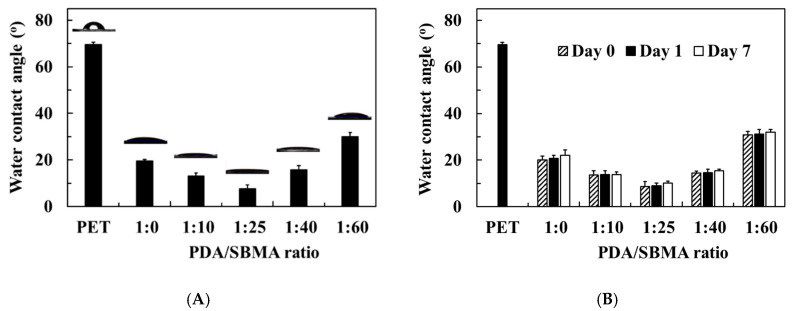
(**A**) Static WCAs of the various PDA/SBMA coatings. (**B**) Surface wettability of the coating for long-term immersion in PBS.

The surface wettability of the PDA/SBMA was further evaluated by WCA, and the results are presented in Figure 5A. The pristine PET has a WCA of approximately 70°. After PDA/SBMA coating via the NaIO_4_ trigger, the WCA of the modified surfaces declined significantly. In the 1:0 coating, the WCA declined to 19.5°, which was attributed to the intrinsic hydrophilicity of PDA. Next, the WCA first decreases and then increases with the increase in SBMA concentration. It reaches the lowest value of 7.5°, indicating super hydrophilicity when the mass ratio of dopamine to SBMA is 1:25. The super hydrophilicity could be attributed to the presence of the most sulfobetaine moieties on the 1:25 surface (XPS data), which enabled a high hydration by electrostatic interaction with water [36,37]. Subsequently, the WCA further increases to 30.0° when the mass ratio of dopamine to SBMA is 1:60. The PDA/SBMA coatings were further immersed in a saltwater bath for one week. We found the WCA kept on original value after the coatings were immersed in a saltwater bath for a long time, meaning the coatings did not peel off from the substrates (Figure 5B). The results indicated the PDA/SBMA coatings were quite stable in the high salt environment.

### 3.3. Antibacterial Properties

**Figure 6:**
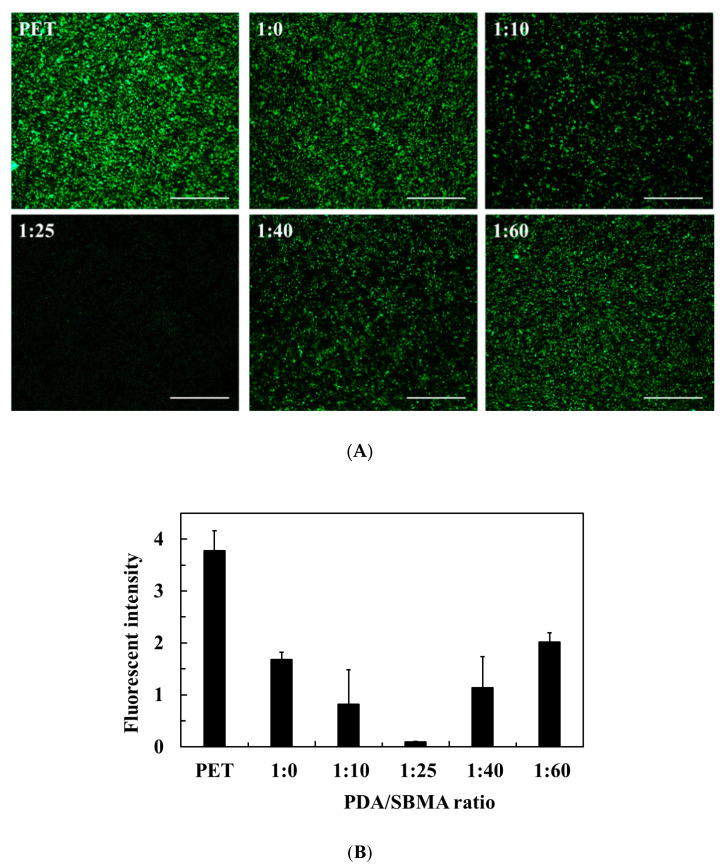
(**A**) Fluorescence images of *E. coli* attachment on the various PDA/SBMA coatings. Scale bar = 100 μm. (**B**) The fluorescence intensity was analyzed from the photographs by Image J. n = 5.

*E. coli* attachment was used to evaluate the non-fouling properties of the coatings. Figure 6 presents the fluorescence photographs reflecting the bacterial attachment on the PDA/SBMA surfaces. The presence of dense small green dots on the PET surfaces indicated that the surface is highly fouling. The fluorescence intensity analyzed from the photographs was calculated to be 3.78. After coating PDA/SBMA in various ratios, the fluorescence intensity was found to depend on the amount of SBMA. As the dopamine-to-SBMA ratio increased from 1:0 to 1:25, the fluorescence intensity decreased gradually and reached a minimum value of 0.09 at 1:25. Subsequently, the fluorescence intensity showed a gradual increase as then SBMA content increased. The fluorescence intensities reached 1.13 and 2.02 at ratios 1:40 and 1:60, respectively. The profile trend between fluorescence intensity and the dopamine-to-SBMA ratio is consistent with that between WCA and the dopamine-to-SBMA ratio. The results clarify the role of the hydrophilicity of PDA/SBMA in enhancing the fouling resistance, especially in superhydrophilic 1:25 coatings.

### 3.4. Applications of PDA/SBMA Coatings

**Figure 7:**
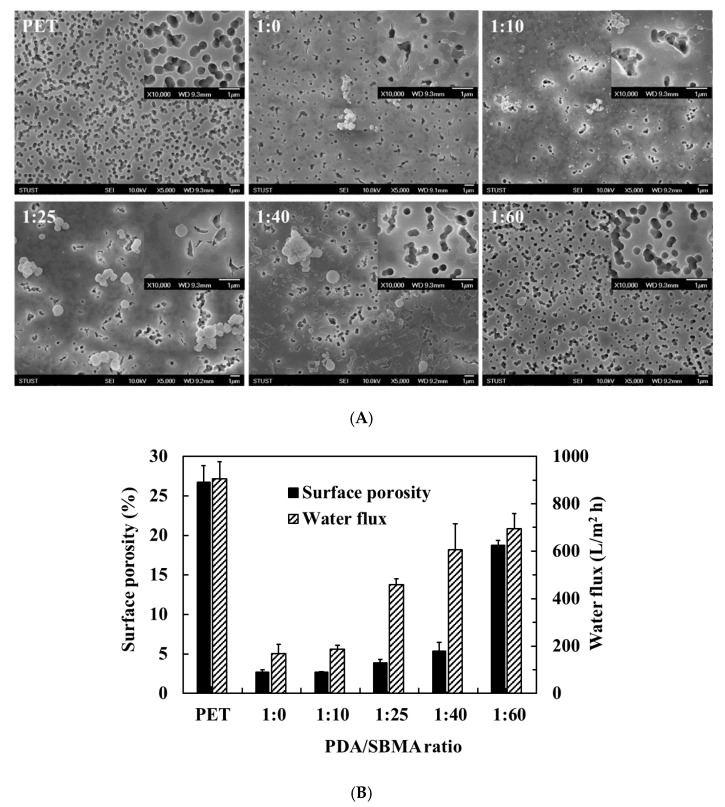
(**A**) SEM images (5000×) and inserts (10,000×) of the various PDA/SBMA coatings on PET membrane. Scale bar = 1 μm. (**B**) Left: surface porosity of membranes as analyzed from SEM images for various PDA/SBMA modified membranes. Right: performance of various PDA/SBMA modified membranes in terms of water flux.

The NaIO_4_ triggered PDA/SBMA coatings on polyester membranes for oil/water separation. First, the surface morphologies of the PDA/SBMA coatings on the membrane were surveyed by SEM (Figure 7A). Observations of the pristine membrane reveal a uniform pore distribution with pore diameters of approximately 0.2 μm, and surface porosity of 26.7%. After the deposition of PDA/SBMA, the pore size shows a significant decrease owing to the formation of PDA/SBMA layers on the membranes. Interestingly, the change in surface porosity depends on the dopamine-to-SBMA ratio. When the dopamine-to-SBMA ratio increases from 1:0 to 1:60, the surface porosity first appears to remain constant at 2.7% and then increases as the SBMA concentration increases. The lowest surface porosity is 2.7% originates from a higher packing density and larger particle size of the polymer on the surface. The largest surface porosity is 18.7% owing to the smaller size of the PDA/SBMA particles and low packing density on the membranes when the ratio of SBMA in the polymer solution increases to 60.

Despite the fact that the PDA/SBMA coatings covered the pores, the irregular tortuous porous structure, and interconnected network of the membrane were well-maintained (amplified SEM pictures). The water flux of various PDA/SBMA membranes was investigated to confirm the filtration ability of the PDA/SBMA coatings. Figure 7B shows a comparison of the pure water flux performances of the pristine, 1:0, 1:10, 1:25, 1: 40, and 1:60 coated membranes under vacuum filtration. As expected, the water flux (approximately 905 L/m^2^ h) of the pristine PET membrane was the highest, while the water flux of the PDA/SBMA membranes were lower than that of the untreated pristine membrane. The water flux of the various PDA/SBMA membranes was found to depend on their pore size. The lowest water flux was observed for the 1:0 and 1:10 membranes, while the 1:60 membrane exhibited a higher water flux. It is worth noting that the superhydrophilic property could enhance the water flux despite the small surface porosity of the 1:25 coated membrane [38].

**Figure 8:**
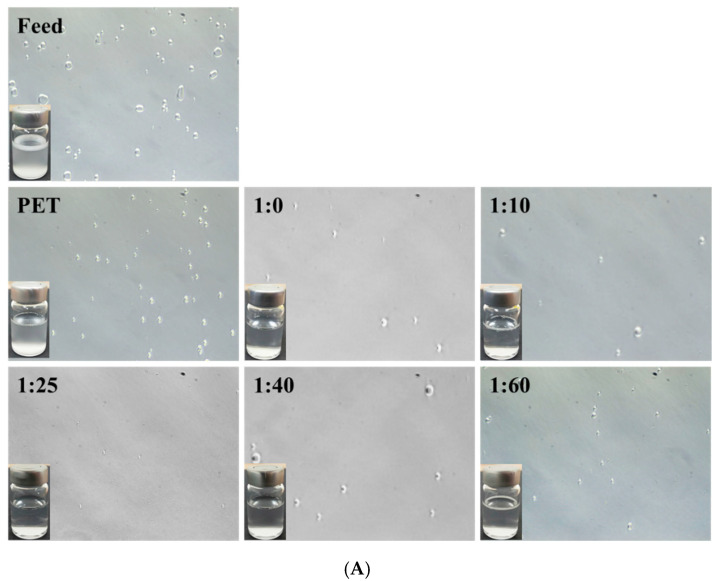

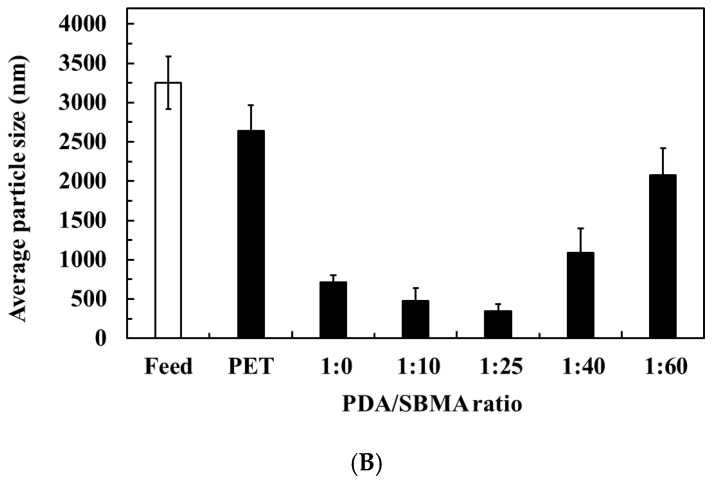
(**A**) Digital images and micrographs of the sodium dodecyl sulfate (SDS) stabilized hexane-in-water emulsion and the filtrate. (**B**) The droplet size of the hexane/water emulsion before and after separation was analyzed by DLS.

The performance of the PDA/SBMA-coated membrane was evaluated by separating an oil–water emulsion. The SDS-stabilized n-hexane-in-water emulsions were prepared and poured into filtration cells to perform filtration separation. Figure 8 shows the presence of many oil drops with a size of about 3.2 μm in the as-prepared hexane/H_2_O emulsion. After filtration through the unmodified membranes, many oil drops were observed. It is worth noting that the amount of oil drops in all collected filtrates decreased after filtration through the PDA/SBMA membranes. In the 1:25 membranes, almost no oil drops were observed in the collected filtrate, while only 0.3 μm of particle size was detected by DSL, indicating satisfactory separation of the emulsion. The superhydrophilic property of the 1:25 surface ensures the formation of a layer of water on the surface of the membrane, thereby avoiding direct contact between the oil and membrane surfaces during oil/water separation. The results clarify the role of the hydrophilicity of PDA/SBMA in enhancing the oil-fouling resistance and successfully separating the oil in the emulsion.

Some recent studies have reported facile methods for preparing polySBMA surfaces via mussel-inspired coatings. For example, Dizon et al. synthesized a novel zwitterionic copolymer containing a catechol group, which can form a stable coating on various materials [39]. However, the method necessitates the synthesis of monomers and copolymers via a complicated reaction. Zhang et al. reported the rapid co-deposition of dopamine and polySBMA triggered by CuSO_4_/H_2_O_2_ [40]. However, this method also requires the prior synthesis of the polymer followed by the co-deposition with dopamine on the surfaces. Although Ma et al. developed a fast approach by mixing the oxidant, dopamine, and zwitterionic monomers to immobilize zwitterionic polymers, the coatings did not exhibit superhydrophilic properties [41]. We compared the existing approach with other mussel-inspired methods for membrane coatings, as shown in Table 2. In can be seen that NaIO_4_ triggered a rapid polymerization as well as obtained the lowest WCA in this study. After 2 h of polymerization, the reactive products (dopaminochrome) did not change significantly when the operating time was increased (Figure S2), indicating the stability of the reactive solution. Therefore, the developed approach has great potential for industrial application.

## 4. Conclusions

We developed a facile and rapid one-step approach for fabricating superhydrophilic surfaces by mixing dopamine and SBMA in the presence of NaIO_4_. After investigations using UV–VIS spectrophotometry and DLS, we found that only the NaIO_4_-triggered PDA solution exhibited the formation of dopaminochrome and the bigger particle sizes of PDA at 2 h of polymerization compared to the UV-triggered solution and without a triggered solution. AFM, XPS and SPR confirms that the PDA/SBMA was successfully bound to the surface and revealed that when the ratio of dopamine to SBMA was 1:25 by weight, one could obtain the most sulfobetaine moieties on the surface, rendering the surface superhydrophilic (WCA < 10°) and antifouling of *E. coli*. The introduction of the superhydrophilic coating to commercial polyester membranes well-maintained the irregular tortuous porous structure and the interconnected network of the membrane, which endowed a great potential for oil/water separation. This one-step modification method using NaIO_4_ as trigger, which has significant advantages over the existing ones for preparing antifouling surfaces, can be potentially implemented in industrial processes.

## Figures and Tables

**Table 1:** Formula, roughness, and element composition of the PDA/SBMA coatings. PDA, polydopamine; SBMA, sulfobetaine methacrylate.

PDA/SBMA Coating	Dopamine (mg/mL)	SBMA (mg/mL)	Roughness (Rs, nm)	Atomic Composition (%)			
				C1s	O1s	N1s	S2p
PET	-	-	3.95	73.0	27	<0.1	<0.1
1:0	2	0	8.22	73.5	19.3	7.2	<0.1
1:10	2	20	6.14	71.8	21.0	6.3	0.9
1:25	2	50	4.32	69.1	22.3	6.7	1.8
1:40	2	80	3.47	68.8	22.9	6.6	1.6
1:60	2	120	5.43	70.3	22.9	5.1	1.6

**Table 2:** The deposition conditions via mussel-inspired modification approaches.

Materials	Oxidations	Coating Time	Substrates	Hydrophilicity	Approach	Ref.
Dopamine, octadecylamine	-	1.5 h + 4 h	Cotton fabrics	Hydrophobic (WCA = 167°)	Two-step	[42]
Dopamine, chitosan	-	24 h + 10 h	Cotton fabrics	Water adsorption in 6 s	Two-step	[43]
Dopamine, polyethyleneimine	-	6, 12, 24 h	Poly(vinylidene fluoride)	Hydrophilic (WCA = 12°~57°)	One-step	[44]
Dopamine, polySBMA	CuSO_4_/H_2_O_2_	1 h	Polypropylene	Hydrophilic (WCA < 20°)	One-step	[40]
Dopamine, SBMA	NaIO_4_	2 h	Polyester	Hydrophilic (WCA < 10°)	One-step	This study

## Supplementary Materials

The following are available online at https://www.mdpi.com/2073-4360/12/9/2008/s1, Figure S1: Particle size of PDA/SBMA solution at 2 h with NaIO_4_ trigger, Figure S2: UV-VIS spectral change for PDA/SBMA (1:25) solution without (A) and with NaIO_4_ (B) trigger against the reaction time. The graph of OD 410 values with respect to the polymerization time. Figure S3: Soybean oil were used for oil/water separation.
