# Supplementary material for: Superhydrophilic Coating with Antibacterial and Oil-Repellent Properties via NaIO4-Triggered Polydopamine/Sulfobetaine Methacrylate Polymerization
Source: Polymers (Basel). 2020 Sep 3;12(9):2008. doi: 10.3390/polym12092008 (PMC7565826; doi:10.3390/polym12092008)

Figure S1 Particle size of PDA/SBMA solution at 2 hr with $NaIO_4$ trigger.

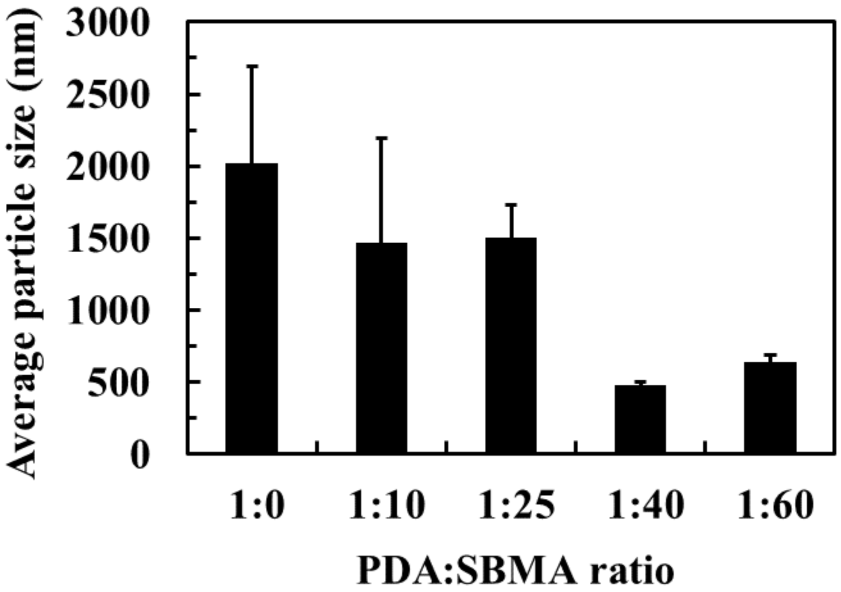

Figure S2 UV-vis spectral change for PDA/SBMA (1:25) solution without (A) and with $NaIO_4$ (B) trigger against the reaction time. The graph of OD 410 values with respect to the polymerization time.

(A) In tris-base

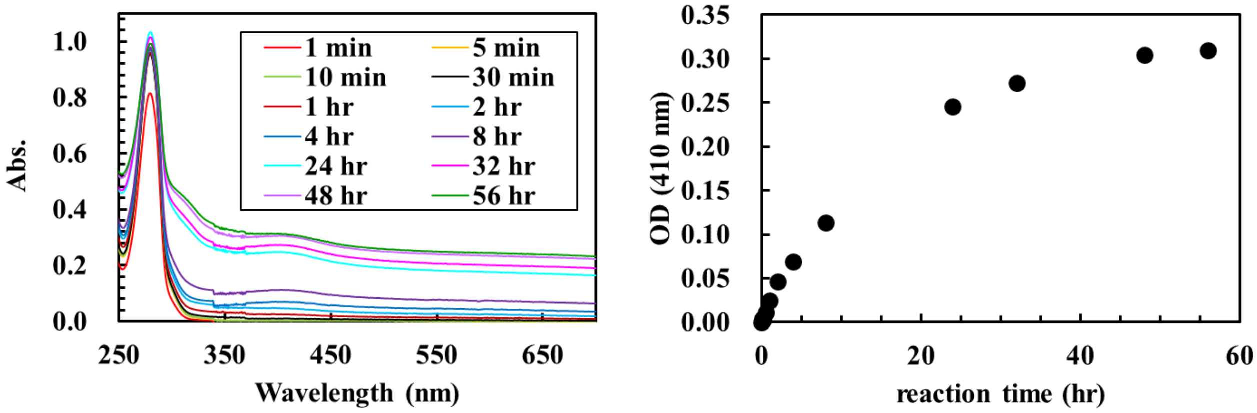

(B) $NaIO_4$ trigger

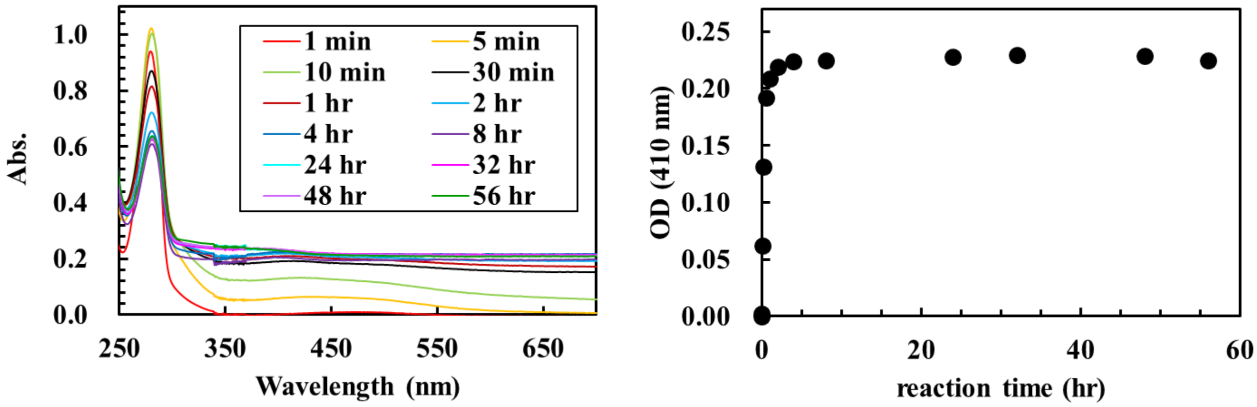

Figure S3 Soybean oil were used for oil/water separation. The filter papers were fixed between the top conical flask and the bottom funnel. The filter papers were wetted by water prior to the separation. The mixture consisted of 20 mL of DI water and 20 mL of soybean oil was poured into the filter to be separated. For the bare PET filter paper, the water in the mixtures immediately flowed through the papers, while the oil stayed above the papers. However, some of oil were observed in the collected filtrate. In contrast to the visible oil droplets passing bare PET filter paper, no visible oil was observed in the filtrate due to the super hydrophilic PDA/SBMA (1:25) coatings.

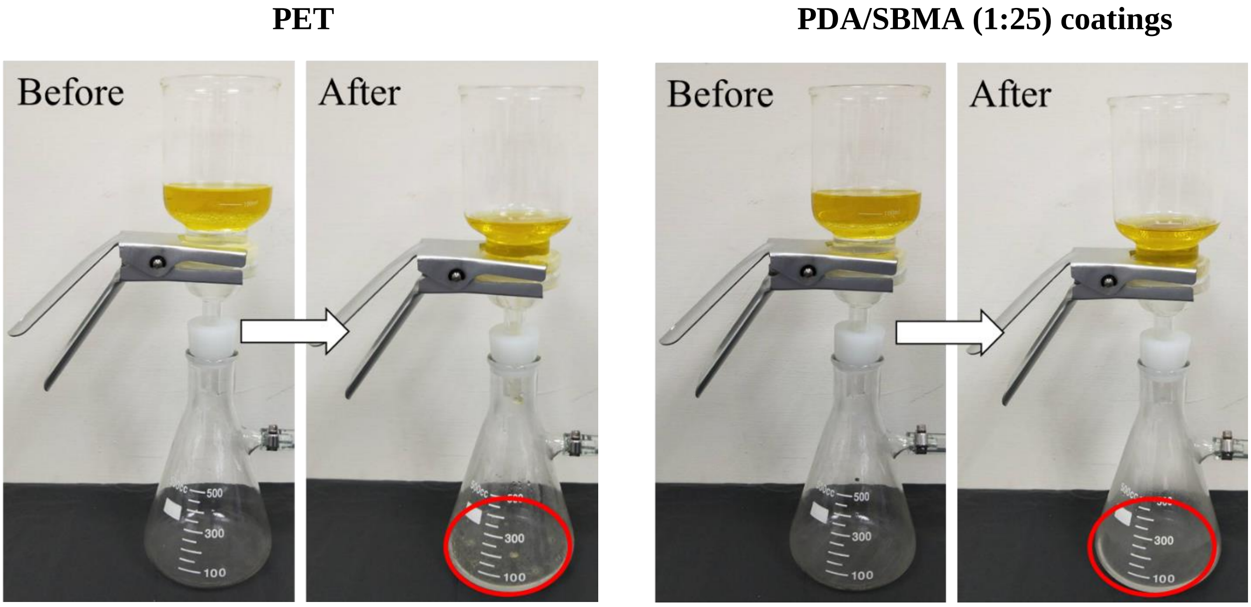

Supplement: Supplementary file 1 [file polymers-12-02008-s001.pdf]
